# Measuring health-related quality of life in colorectal cancer patients: systematic review of measurement properties of the EORTC QLQ-CR29

**DOI:** 10.1007/s00520-019-04764-7

**Published:** 2019-04-13

**Authors:** Anja van der Hout, Koen I. Neijenhuijs, Femke Jansen, Cornelia F. van Uden-Kraan, Neil K. Aaronson, Mogens Groenvold, Bernhard Holzner, Caroline B. Terwee, Lonneke V. van de Poll-Franse, Pim Cuijpers, Irma M. Verdonck-de Leeuw

**Affiliations:** 1 Department of Clinical, Neuro- and Developmental Psychology, Faculty of Behavioral and Movement Sciences, Amsterdam Public Health institute, Vrije Universiteit Amsterdam, Van der Boechorststraat 7, 1081 BT Amsterdam, The Netherlands; 2 Cancer Center Amsterdam (CCA), VU University Medical Center, De Boelelaan 1118, 1081 HV Amsterdam, The Netherlands; 3 Division of Psychosocial Research and Epidemiology, The Netherlands Cancer Institute, Plesmanlaan 121, 1066 CX Amsterdam, The Netherlands; 4 The Research Unit, Department of Palliative Medicine, Bispebjerg Hospital, Copenhagen University Hospital, 20D, Bispebjerg Bakke 23, NV 2400 Copenhagen, Denmark; 5 Department of Public Health, University of Copenhagen, Øster Farimagsgade 5, 1014 Copenhagen, Denmark; 6 Department of Psychiatry, Psychotherapy and Psychosomatics, Medical University of Innsbruck, Anichstraβe 35, 6020 Innsbruck, Austria; 7 Department of Epidemiology and Biostatistics, Amsterdam Public Health institute, VU University Medical Center, De Boelelaan 1089a, 1081 HV Amsterdam, The Netherlands; 8 The Netherlands Comprehensive Cancer Organisation, Godebaldkwartier 419, 3511 DT Utrecht, The Netherlands; 9 CoRPS - Center of Research on Psychology in Somatic diseases, Department of Medical and Clinical Psychology, Tilburg University, Warandelaan 2, 5037 AB Tilburg, The Netherlands; 10 Department of Otolaryngology – Head and Neck Surgery, VU University Medical Center, De Boelelaan 1117, 1081 HV Amsterdam, The Netherlands

**Keywords:** EORTC QLQ-CR29, Patient-reported outcome measure (PROM), Health-related quality of life, Colorectal cancer, Systematic review, Measurement property

## Abstract

**Introduction:**

The EORTC QLQ-CR29 is a patient-reported outcome measure to evaluate health-related quality of life among colorectal cancer patients in research and clinical practice. The aim of this systematic review was to investigate whether the initial positive results regarding the measurement properties of the QLQ-CR29 are confirmed in subsequent studies.

**Methods:**

A systematic search of Embase, Medline, PsycINFO, and Web of Science was conducted to identify studies investigating the measurement properties of the QLQ-CR29 published up to January 2019. For the 11 included studies, data were extracted, methodological quality was assessed, results were synthesized, and evidence was graded according to the COnsensus-based Standards for the selection of health Measurement INstruments (COSMIN) methodology on the measurement properties: structural validity, internal consistency, reliability, measurement error, construct validity (hypothesis testing, including known-group comparison, convergent and divergent validity), cross-cultural validity, and responsiveness.

**Results:**

Internal consistency was rated as “sufficient,” with low evidence. Reliability was rated as “insufficient,” with moderate evidence. Construct validity (hypothesis testing; known-group comparison, convergent and divergent validity) was rated as “inconsistent,” with moderate evidence. Structural validity, measurement error, and responsiveness were rated as “indeterminate” and could therefore not be graded.

**Conclusion:**

This review indicates that current evidence supporting the measurement properties of the QLQ-CR29 is limited. Additionally, better quality research is needed, taking into account the COSMIN methodology.

**Electronic supplementary material:**

The online version of this article (10.1007/s00520-019-04764-7) contains supplementary material, which is available to authorized users.

## Introduction

Colorectal cancer (CRC) is among the most prevalent cancers worldwide [1]. CRC and its treatment can have a large impact on health-related quality of life (HRQOL) [2]. It is important to assess HRQOL in clinical trials to investigate the impact of a treatment on HRQOL, and in clinical practice to detect and monitor symptoms and offer optimal care [3–5]. A frequently used patient-reported outcome measure (PROM) to evaluate HRQOL in cancer patients is the 30-item European Organization for Research and Treatment of Cancer (EORTC) Quality of Life Core Questionnaire (EORTC QLQ-C30) [6, 7] and its tumor-specific questionnaire modules [8]. In 1999, the 38-item module for CRC patients was developed (EORTC QLQ-CR38) [9], and in 2007, the module was revised and shortened to 29 items (EORTC QLQ-CR29) [10]. The initial validation study of the QLQ-CR29 in an international sample of CRC patients [11] showed that it had good internal consistency (α > 0.70) in all but one subscale, was acceptably reliable (intraclass correlation coefficient (ICC) > 0.68 for subscales and > 0.55 for single items), was able to discriminate between known groups (patients with and without stoma, Karnofsky performance score < 80 and > 80, and with curative and palliative treatment), and had good divergent validity, i.e., low correlation with QLQ-C30 items [11].

Two systematic reviews on the measurement properties of the QLQ-CR29 were published in 2015 and 2016 [12, 13]. Wong et al. performed a systematic review on various disease-specific and generic HRQOL PROMs, and included two studies on the QLQ-CR29. They recommended the QLQ-CR38 to assess HRQOL in CRC patients, because it had the most positive ratings on the measurement properties according to their quality assessment criteria [12]. Ganesh et al. performed a systematic review of three CRC-specific PROMs (Functional Assessment of Cancer Therapy-Colorectal (FACT-C), QLQ-CR38, and QLQ-CR29), and included three studies on the QLQ-CR29. They concluded that the choice for one of these three instruments depends on the context and the research aim [13].

Since these reviews, several new validation studies of the QLQ-CR29 have been published. Therefore, the aim of the present study was to perform a systematic review of the measurement properties of the QLQ-CR29 as investigated in validation studies up to 2019, according to the COnsensus-based Standards for the selection of health Measurement INstruments (COSMIN) criteria [14, 15], and to investigate whether the initial positive results regarding the measurement properties of the QLQ-CR29 are confirmed.

## Materials and methods

### EORTC QLQ-CR29

The EORTC QLQ-CR29 is a tumor-specific HRQOL questionnaire module for CRC patients, which is designed to complement the EORTC QLQ-C30 questionnaire [6, 10]. The QLQ-CR29 has five functional and 18 symptom scales. It contains four subscales (urinary frequency (UF), blood and mucus in stool (BMS), stool frequency (SF), and body image (BI)) and 19 single items (urinary incontinence, dysuria, abdominal pain, buttock pain, bloating, dry mouth, hair loss, taste, anxiety, weight, flatulence, fecal incontinence, sore skin, embarrassment, stoma care problems, sexual interest (men), impotence, sexual interest (women), and dyspareunia) [11]. Patients are asked to indicate their symptoms during the past week(s). Scores can be linearly transformed to provide a score from 0 to 100. Higher scores represent better functioning on the functional scales and a higher level of symptoms on the symptom scales [10, 11].

### Literature search

The literature search was part of a larger systematic review (Prospero ID 42017057237) [16], investigating the validity of 39 PROMs measuring HRQOL of cancer patients included in an eHealth self-management application “Oncokompas” [17–21]. We performed a systematic search of Embase, Medline, PsycINFO, and Web of Science, to identify studies investigating the measurement properties of these 39 PROMs, including the QLQ-CR29. The search terms were the PROM’s name, combined with search terms for cancer, and a precise filter for measurement properties [22]. The full search terms can be found in Appendix. The literature search was performed in June 2016 and updated in January 2019 to search for recent studies on the QLQ-CR29 specifically. References of included studies have been checked for additional articles manually.

### Inclusion and exclusion criteria

Inclusion criteria were as follows: reporting original data from cancer patients on at least one measurement property of the QLQ-CR29, as defined in the COSMIN taxonomy [15, 23, 24]—structural validity (the degree to which the scores of a PROM are an adequate reflection of the dimensionality of the construct to be measured), internal consistency (the degree of interrelatedness among items), reliability (the extent to which scores of patients who have not changed are the same for repeated measures on different occasions), measurement error (the error of a patient’s score that is not attributed to true changes in the construct to be measured), construct validity (hypothesis testing; including known-group comparison, convergent and divergent validity [the degree to which the scores of a PROM are consistent with hypotheses with regard to differences between relevant groups, and relationships to scores of other instruments, respectively]), cross-cultural validity (the degree to which the performance of the items on a translated or culturally adapted PROM are an adequate reflection of the performance of the items of the original version of the PROM), and responsiveness (the ability of a PROM to detect change over time in the construct to be measured) [24]. Exclusion criteria were as follows: no availability of full-text manuscripts, conference proceedings, and non-English publications. Titles and abstracts, and eligible full texts were screened by two of the four raters independently (KN, FJ, AH, NH). Disagreements were discussed until consensus was reached.

### Data extraction

For each reported measurement property defined by the COSMIN taxonomy [25], data were extracted by two of the four extractors independently (KN, FJ, AH, NH). This included type of measurement property, its outcome, and information on methodology. Disagreements were discussed until consensus was reached.

### Data synthesis

For the data synthesis, we followed the three steps of the COSMIN guideline for systematic review of PROMs [26]. In step 1, we rated methodological quality of the studies per reported measurement property as either “excellent,” “good,” “fair,” or “poor.” A total score was obtained by taking the lowest rating on any of the methodological aspects, according to the original COSMIN checklist [14]. In step 2, we rated the results per measurement property, by applying the COSMIN criteria for good measurement properties [26]. Results of the individual studies were rated as “sufficient,” “insufficient,” or “indeterminate” per measurement property, according to predefined criteria. Ratings from the individual studies were then qualitatively summarized into an overall rating per measurement property. Inconsistencies between studies were explored. If any explanation was found (e.g., poor methodological quality), this was taken into account in the overall rating, if no explanation was found, the overall rating was summarized as “inconsistent.” In step 3, we graded the quality of the evidence of the measurement properties, following a modified GRADE approach [26]. The overall quality of the evidence was rated as “high,” “moderate,” “low,” or “very low,” taking into account risk of bias, inconsistency of study results, imprecision, and indirectness. When a measurement property was rated as “indeterminate” in step 2, the quality of evidence could not be graded, as there was no evidence to grade. The evaluation of measurement properties was performed by two raters independently (AH, NH). Disagreements were discussed until consensus was reached.

## Results

In the initial search, 980 nonduplicate abstracts were identified for all 39 PROMs, of which 31 were relevant for the QLQ-CR29. The search update resulted in 27 extra nonduplicate abstracts regarding the QLQ-CR29. In total, 55 abstracts were screened, of which 30 were excluded. Thirteen studies were excluded during full-text screening. One study was excluded during data extraction [27], because data were not presented for the QLQ-CR29 separately. The flow diagram of the literature search and selection process is shown in Supplementary Fig. 1. Study characteristics of the 11 included studies [11, 28–37] are shown in Table 1. These studies reported on structural validity (9 studies), internal consistency (10 studies), reliability (6 studies), construct validity (know-group comparison [10 studies], convergent [8 studies], and divergent [2 studies] validity), and responsiveness (2 studies), but did not report on measurement error, or cross-cultural validity. However, measurement error could be calculated for four studies.

**Table 1:** Study characteristics of the included studies

Reference	Year of publication	Research aim	Population	Sample size	Language	Characteristic of study sample
Arraras et al. [28]	2011	Validation of QLQ-CR29	Spanish rectal cancer patients	84	Spanish	Sex, 67% male Stoma, 29% yes Tumor type, 100% rectal cancer Age, 65.2 ± 9.5 (44–82)
Hou et al. [29]	2015	Validation of Low Anterior Resection Syndrome (LARS) score	Chinese rectal cancer patients	102	Chinese	Sex, 58% male Stoma, 22% yes Tumor type, 100% rectal cancer Age, 66.5 ± 10.7 (37–86)
Ihn et al. [30]	2015	Validation of QLQ-CR29	Korean CRC patients	123	Korean	Sex, 69% male Stoma, not reported Tumor type, 50% CRC, 50% rectal cancer Age, 60.1 ± 9.6
Lin et al. [31]	2017	Validation of QLQ-CR29	Chinese CRC patients	356	Chinese (Simplified Chinese)	Sex, 63% male Stoma, not reported Tumor type, 56% CRC, 43% rectal cancer, 1% both Age, 54.5 ± 13.5
Magaji et al. [32]	2015	Validation of QLQ-CR29	Malaysian CRC patients	93	Bahasa Malaysian	Sex, 59% male Stoma, 34% yes Tumor type, 52% CRC, 38% rectal cancer, 10% unknown Age, not reported
Montazeri et al. [33]	2018	Validation of QLQ-CR29	Iranian CRC patients	100	Persian	Sex, 47% male Stoma, 33% Tumor type, not reported Age, 53.6 ± 12.6 (22–78)
Nowak et al. [34]	2011	Validation of QLQ-CR29 (pilot)	Polish rectal cancer patients	20	Polish	Sex, 50% male Stoma, 50% yes Tumor type, 100% rectal cancer Age, not reported
Sanna et al. [35]	2017	Validation of QLQ-CR29	Polish CRC patients	150	Polish	Sex, 61% male Stoma, 30% yes Tumor type, 61% CRC, 39% rectal cancer Age, 68 ± 12.5 (32–85)
Shen et al. [36]	2018	Validation of QLQ-CR29	Taiwanese CRC patients	108	Traditional Chinese (Mandarin)	Sex, 58% male Stoma, 10% Tumor type, 64% CRC, 36% rectal cancer Age, 63.7 ± 13.2 (22–89)
Stiggelbout et al. [37]	2015	Validation of QLQ-CR29	Dutch CRC patients	236	Dutch	Sex, 61% male Stoma, 29% yes Tumor type, not reported Age, 65 ± 11.3 (24–90)
Whistance et al. [11]	2009	Validation of QLQ-CR29 (original validation)	International population of CRC patients	351	Spanish, English, French, Taiwanese, Italian, German	Sex, 58% male Stoma, 33% yes Tumor type, 56% CRC, 44% rectal cancer, 1% unknown Age, 65.0 ± 11.9

### Structural validity

Nine studies investigated *structural validity* (Table 2). Methodological quality of eight studies was rated as “poor” [11, 28, 30–32, 34–36], because they performed multitrait item scaling (MIS) instead of exploratory/confirmatory factor analysis (EFA/CFA). One study was rated as “fair” [37], because it performed a principal component analysis (PCA). The studies reporting MIS were consistent in their findings, showing no inconsistent items regarding convergent and discriminant validity within the subscale. However, since MIS is an indirect test of structural validity, no conclusions can be drawn on the basis of these studies. In the study that used PCA, three of the four original subscales (UF, BMS, and BI) were replicated. The two-item original SF subscale was merged with four single items about bowel or stoma problems into a new subscale “defecation/stoma problems (DSP)” [37]. Because the account of variability and the ratio of the explained variance by the factors was not reported, the PCA cannot be interpreted properly, and therefore *structural validity* was rated as “indeterminate,” and there is no evidence for or against unidimensionality of the subscales.

**Table 2:** Structural validity of the EORTC QLQ-CR29

Reference	Methodology	Results	Quality	Rating
Arraras et al. [28]	MIS	Most items exceeded correlations of 0.4 with other items in their own subscale, except for items 38 and 39 (BMS subscale). All items had a higher correlation with other items in their own scale than with items in other subscales, except for item 38 (blood in stool).	Poor	Indeterminate
Ihn et al. [30]	MIS	All items exceeded correlations of 0.4 with other items in their own subscale, for the total population and for patients with and without stoma. All items had a higher correlation with other items in their own subscale, than with items in other subscales, for the total population and for patients with and without stoma.	Poor	Indeterminate
Lin et al. [31]	MIS	All items exceeded correlations of 0.4 with other items in their own subscale. All item had a higher correlation with other items in their own subscale than with items in other subscales.	Poor	Indeterminate
Magaji et al. [32]	MIS	All items exceeded correlations of 0.4 with other items in their own subscale, for the total population and for patients with and without stoma. All items had a higher correlation with other items in their own subscale than with items in other subscales, for the total sample, as well as patients with and without stoma.	Poor	Indeterminate
Nowak et al. [34]	MIS	Most items exceeded correlations of 0.4 with other items in their own subscale, for the total population. For the BMS subscale, convergent and divergent validity could not be estimated for the total population. In subgroups of patients with and without stoma, mixed results with also negative correlations were shown. Most items had a higher correlation with items in other subscales, than with items in their own subscale, for the total population and for patients with and without stoma.	Poor	Indeterminate
Sanna et al. [35]	MIS	All items exceeded correlations of 0.4 with other items in their own subscale for the total population and for patients with and without stoma. All items had a higher correlation with other items in their own subscale than with items in other subscales, for the total population and for patients with and without stoma.	Poor	Indeterminate
Shen et al. [36]	MIS	All items exceeded correlations of 0.4 with other items in their own subscale. All items had higher correlations with other items in their own subscale than with items in other subscales.	Poor	Indeterminate
Stiggelbout et al. [37]	PCA	Seven factors were revealed, of which three of the original subscales were reproduced. The two-item original factor SF was combined with all items about bowel and stoma problems into a new six-item subscale, Defaecation/Stoma Problems (DSP). All remaining factors did not form clearly interpretable subscales.	Fair	Indeterminate
Whistance et al. [11]	MIS	All items exceeded correlations of 0.4 with other items in their own subscale, for the total sample, as well as for patients with and without stoma, except for the BMS subscale in patients with a stoma (0.37). All items had a higher correlation with other items in their own subscale than with items in other subscales, for the total sample, as well as patients with and without stoma.	Poor	Indeterminate

*MIS* multitrait item scaling analysis, *PCA* principal component analysis

### Internal consistency

Ten studies investigated *internal consistency* (Supplementary Table 1). Methodological quality of nine studies was rated as “poor” [11, 28, 30–36], because evidence for unidimensionality of the subscales was not provided, and therefore, the value of Cronbach’s α could not be interpreted properly [38]. One study was rated as “fair” [37], because it did not report on how missing items were handled. This study reported good internal consistency for the BI subscale (α = 0.80), and the new subscale established (see “Structural validity”); DSP (α = 0.84). The subscale UF had adequate internal consistency (α = 0.71), and for the original subscale SF two values were presented; for patients with (α = 0.72) and without stoma (α = 0.68). The subscale BMS had low internal consistency (α = 0.56) [37]. The studies of poor quality showed mostly adequate Cronbach’s α values, except for the BMS subscale. Based on these findings, the evidence on *internal consistency* was rated “sufficient,” because > 75% of the values were good for the original subscales, assuming these subscales are unidimensional, which is not proven with PCA (see “Structural validity”). Quality of evidence was graded as “low,” because only one study of fair quality was found.

### Reliability

Six studies investigated test–retest *reliability* (Table 3). Methodological quality of two studies was rated as “poor” [36, 37], because of the small sample size. Four studies were rated as “fair” [11, 30, 32, 35], because it was not reported how missing items were handled and/or had a moderate sample size. Two of these studies [11, 32] provided an overall ICC value for all subscales/items with exceptions (e.g., “ICC for all subscales was > 0.66, except for BI”), and thereby provided too little information to interpret the ICC on the subscale/item level. Low correlations (< 0.70) were reported in two remaining studies for the UF subscale, and in one of the two studies for multiple single items [30, 35]. Based on these findings, evidence on *reliability* was rated as “insufficient,” because of multiple unacceptable ICC values across studies. Quality of evidence was graded as “moderate,” because only studies of fair and poor quality were found.

**Table 3:** Test–retest reliability (correlation coefficients) of the of QLQ-CR29

Reference	UF	BMS	SF	BI	DSP	UI	DY	AP	BP	BF	DM	HL	TA	ANX	WEI	FL	FI	SS	EMB	STO	IMP	DYS	SEXM	SEXW	Quality	Rating
Ihn et al. [30]	0.64	–	0.89	0.92	–	1.00	0.92	0.85	0.94	0.78	0.76	0.95	0.78	0.83	0.96	0.69	0.79	0.93	0.81	0.86	0.67	–	0.79	–	Fair	Sufficient
Magaji et al. [32]	0.45	> 0.51	> 0.51	> 0.51	–	> 0.51	> 0.51	> 0.51	0.49	> 0.51	> 0.51	0.14	> 0.51	0.30	> 0.51	> 0.51	> 0.51	> 0.51	> 0.51	0.41	> 0.51	0.33	> 0.51	> 0.51	Fair	Insufficient
Sanna et al. [35]	0.59	0.88	0.90	0.91	0.89	0.41	0.47	0.85	0.89	0.85	0.81	0.74	0.79	0.70	0.82	0.82	0.80	0.88	0.81	–	0.80	0.77	0.82	0.81	Fair	Sufficient
Shen et al. [36]	0.51	0.34	0.78	0.89	–	0.11	0.69	0.68	0.71	0.40	0.09	0.85	0.67	0.47	0.48	0.81	0.47	0.63	0.50	0.89	0.97	0.65	0.47 ^b^	0.47 ^b^	Poor	Insufficient
Stiggelbout et al. [37]	0.33–0.43^a^	0.72–0.90^a^	0.27–0.81^a^	0.41–0.76^a^	–	0.20	0.36	0.79	0.74	0.55	0.93	0.82	0.75	0.54	0.71	0.64	0.75	0.82	0.65	–	0.78 ^b^	0.78 ^b^	0.85 ^b^	0.85 ^b^	Poor	Insufficient
Whistance et al. [11]	> 0.68	> 0.68	> 0.68	> 0.68	–	> 0.55	> 0.55	> 0.55	> 0.55	> 0.55	> 0.55	> 0.55	> 0.55	> 0.55	> 0.55	> 0.55	> 0.55	> 0.55	> 0.55	> 0.55	> 0.55	> 0.55	> 0.55	> 0.55	Fair	Indeterminate

Subscales: UF = urinary frequency; BMS = blood and mucus in stool; SF = stool frequency; BI = body image; DSP = defecation/stoma problemsSingle items: UI = urinary incontinence; DY = dysuria; AP = abdominal pain; BP = buttock pain; BF = bloating; DM = dry mouth; HL = hair loss; TA = taste; ANX = anxiety; WEI = weight; FL = flatulence; FI = fecal incontinence; SS = sore skin; EMB = embarrassment; STO = stoma care problems; IMP = impotence; DYS = dyspareunia; SEXM = sexual interest (men); SEMW = sexual interest (women)

^a^Range of ICCs of single items within the subscale

^b^ICCs are combined for men and women

### Measurement error

None of the studies reported on *measurement error*. However, standard error of measurement (SEM) and smallest detectable change (SDC) could be calculated for four studies reporting on test–retest reliability [30, 35–37], using the ICCs and standard deviations of the subscales and single items (Supplementary Table 2). Methodological quality of two studies was rated as “fair” [30, 35], because of the moderate sample size. Two studies were rated as “poor” [36, 37], because of the small sample size. SDC scores ranged between 9.41 and 54.21, representing 9–54% of the scale of the QLQ-CR29 (0–100). However, because the minimal important change (MIC) was not reported, measurement error could not be interpreted. Based on these findings, the evidence on *measurement error* was rated as “indeterminate.”

### Construct validity (hypothesis testing)

#### Known-group comparison

Ten studies performed a *known-group comparison*; a comparison of subgroups based on sociodemographic and/or clinical variables where differences in QLQ-CR29 scores should be expected (Table 4). Methodological quality of eight studies was rated as “poor” [11, 30–35, 37], because they did not formulate a priori hypotheses about expected differences between groups. Two studies were rated as “fair” [28, 36], because it was not described how missing items were handled. The studies of fair quality showed multiple differences in subscales and items between known-groups, but confirmed less than 75% of their hypotheses, leading to an “insufficient” rating. The studies of poor quality found multiple differences between groups (e.g., difference in taste for stoma vs. no stoma group), but careful interpretation is warranted, because no hypotheses were formulated, therefore leading to an “indeterminate” rating.

**Table 4:** Known group comparison of the QLQ-CR29

Reference	Comparison groups	Outcome ^a^	Quality	Rating
Arraras et al. [28]			Fair	Insufficient
	Age (45–65 vs. 66–82 years)	Older patients (66–82 years) had significantly lower functioning scores related to sexual interest (men), higher symptom scores related to taste, and lower symptom scores related to dyspareunia (women), compared to younger patients (45–65 years).		
	Limiting comorbidity (yes vs. no)	Patients with limiting comorbidity had significantly lower functioning scores related to sexual interest (men), and higher symptom scores related to impotence (men), compared to patients without limiting comorbidity.		
	Performance status (Karnofsky score ≤ 90 vs. > 90)	Patients with a lower performance status (KPS ≤ 90) had significantly higher symptom scores related to blood and mucus in stool, compared to patients with a higher performance status (KPS > 90).		
	Adjuvant chemotherapy (yes vs. no)	Patients who received chemotherapy had significantly lower symptom scores related to taste, compared to patients who did not receive chemotherapy.		
	Type of surgery and presence of stoma (low anterior resection vs. abdominoperineal resection)	Patients with low anterior resection surgery had significantly higher symptom scores related to stool frequency, compared to patients with abdominoperineal resection surgery.		
Ihn et al. [30]			Poor	Indeterminate
	Cancer type (rectal cancer vs. colon cancer)	Patients with rectal cancer had significantly lower functioning scores related to body image, anxiety and weight, and higher symptom scores related to urinary incontinence, abdominal pain, buttock pain, hair loss, taste, flatulence, fecal incontinence, sore skin, embarrassment and impotence (men), compared to patients with colon cancer.		
	Neoadjuvant therapy (yes vs. no)	Rectal cancer patients who had neoadjuvant therapy had significantly lower functioning scores related to body image and anxiety, compared to rectal cancer patients who did not have neoadjuvant therapy.		
	Stoma (yes vs. no)	Rectal cancer patients with a stoma had significantly lower functioning scores related to body image, anxiety and weight, and higher symptom scores related to sore skin and embarrassment, compared to rectal cancer patients without stoma.		
Lin et al. [31]			Poor	Indeterminate
	Stoma (yes vs. no)	Patients with a stoma had significant lower functioning scores related to body image, anxiety, and weight, and higher symptom scores related to urinary incontinence, buttock pain, dry mouth, flatulence, fecal incontinence, sore skin and embarrassment, and lower symptom scores related to taste, compared with patients without a stoma.		
	Performance status (Karnofsky score ≤ 80 vs. > 80)	Patients with a lower performance status (KPS ≤ 80) had significant higher functioning scores related to anxiety, weight, sexual interest (men) and sexual interest (women), lower symptom scores related to urinary frequency, dysuria, buttock pain, hair loss, flatulence, fecal incontinence, sore skin, embarrassment, impotence (men) and dyspareunia (women), and higher symptom scores related to blood and mucus in stool, stool frequency, bloating and taste, compared with patients with a higher performance status (KPS > 80).		
Magaji et al. [32]			Poor	Indeterminate
	Stoma (yes vs. no)	Patients with a stoma had significantly higher symptom scores related to blood and mucus in stool, flatulence, fecal incontinence, sore skin, and embarrassment, compared with patients without a stoma.		
	Performance status (Karnofsky score ≤ 80 vs. > 80)	No significant differences were found between patients with lower (KPS ≤ 80) and higher (KPS > 80) performance status.		
Montazeri et al. [33]			Poor	Indeterminate
	Stoma (yes vs. no)	Patients with a stoma had significantly higher symptoms scores related to urinary frequency, blood and mucus in stool, stool frequency, urinary incontinence, abdominal pain, buttock pain, bloating, flatulence, fecal incontinence, sore skin, embarrassment, and dyspareunia, compared with patients without a stoma.		
Nowak et al. [34]			Poor	Indeterminate
	Sex (males vs. females)	No significant differences were found between men and women.		
	Stoma (yes vs. no)	Patients with a stoma had a significant lower (median) functioning scores related to body image, lower symptom scores related to flatulence, and higher symptom scores related to abdominal pain, compared with patients without stoma.		
Sanna et al. [35]			Poor	Indeterminate
	Stoma (yes vs. no)	Patients with a stoma had significant higher functioning scores related to body image, lower functioning scores related to sexual interest (women), and higher symptom scores related to urinary incontinence, abdominal pain, buttock pain, impotence (men), and dyspareunia (women), compared with patients without stoma.		
	Age (< 65 years vs. ≥ 65 years)	Older patients (≥ 65 years) had significant lower functioning scores related to sexual interest (men) and sexual interest (women), higher symptom scores related to urinary frequency, dry mouth, taste, embarrassment (stoma patients), and stoma care problems (stoma patients), and lower symptom scores related to bloating and dyspareunia (women), compared with younger patients (< 65 years).		
	Treatment intent (Curative vs. palliative)	Patients treated with curative intent had significant higher functioning scores related to sexual interest (men), lower functioning scores related to anxiety, higher symptom scores related to buttock pain, flatulence (patients without stoma), stool frequency (patients without stoma), fecal incontinence (patients with stoma), and lower symptom scores related to hair loss and taste, compared with patients treated with palliative intent.		
Shen et al. [36]			Fair	Insufficient
	Treatment (active treatment vs. follow-up)	Patients with active treatment had a significant higher symptom score related to blood and mucus in stool, compared with patients during follow-up.		
	ECOG status score (ECOG = 0 vs. ECOG = 1–3)	Patients with a high performance status (ECOG = 0) had a significant higher symptom score related to urinary frequency, compared with patients with a lower performance status (ECOG = 1–3).		
	Bristol Stool Scale (BSS) (BSS = 0–4 vs. BSS = 5–6)	No significant differences were found between patients with diarrhea (BSS = 5–6) and without diarrhea (BSS = 0–4).		
	Stoma (yes vs. no)	Patients with a stoma had significant higher symptom scores related to fecal incontinence and sore skin, compared to patient without a stoma.		
	Surgery (minimally invasive vs. laparotomy)	Patients with a minimally invasive surgery had a significant lower symptom score related to buttock pain, compared to patients with a laparotomy.		
	Adjuvant therapy (yes vs. no)	Patient with adjuvant therapy had a significant higher symptom score related to hair loss, compared to patients without adjuvant therapy.		
Stiggelbout et al. [37]			Poor	Indeterminate
	Age (≤ 65 years vs. ≥ 66 years)	Older patients (≥ 66 years) had significantly lower functioning scores related to sexual interest (men) and sexual interest (women), and higher symptom scores related to urinary frequency, urinary incontinence and dry mouth, compared with younger patients (≤ 65 years).		
	Stoma (yes vs. no)	Patients with a stoma had significantly higher functioning scores related to body image and weight, and higher symptom scores related to urinary incontinence, buttock pain and impotence (men), compared with patients without a stoma.		
	Treatment intent (curative vs. palliative)	Patients treated with curative intent had significantly higher symptom scores related to blood and mucus in stool and buttock pain, and lower symptom scores related to hair loss and taste, compared with patients treated with palliative intent.		
Whistance et al. [11]			Poor	Indeterminate
	Stoma (yes vs. no)	Patients with a stoma had significantly higher functioning scores related to body image, and higher symptom scores related to urinary frequency, urinary incontinence, fecal incontinence, sore skin and embarrassment, compared with patients without stoma.		
	Performance status (Karnofsky score < 80 vs. > 80)	Patients with a lower performance status (KPS < 80) had significantly higher symptom scores related to stool frequency, abdominal pain, bloating, dry mouth and flatulence, compared with patients with a higher performance status (KPS > 80).		
	Treatment intent (curative vs. palliative)	Patients treated with curative intent had significantly higher symptom scores related to dyspareunia (women), and lower symptom scores related to hair loss, compared with patients treated with palliative intent.		

^a^Higher functional scores = better functioning, higher symptom scores = more problems

#### Convergent validity

Eight studies investigated *convergent validity* (Table 5). Methodological quality of seven studies were rated as “poor” [28, 30–33, 35, 37], because a priori hypotheses about expected correlations were not reported, and/or information on the measurement properties of the comparator instrument was not provided. One study was rated as “good” [29]. In this study, the comparator instrument was the low anterior resection syndrome (LARS) score (measuring bowel dysfunction after sphincter-preserving surgery among rectal cancer patients [29, 39]). All five of the a priori formulated hypotheses were confirmed, leading to a “sufficient” rating. In the studies of poor quality, the comparator instrument was the QLQ-C30. The QLQ-C30 is the core questionnaire of the EORTC QLQ questionnaires [6]. Most studies showed that functional subscales of the QLQ-CR29 were positively correlated with functional scales of the QLQ-C30, and negatively correlated with symptom scales of the QLQ-C30, and that most QLQ-CR29 symptom scales were positively correlated with symptom scales of the QLQ-C30, and negatively correlated with functional scales of the QLQ-C30. As there were no a priori hypotheses reported in most of these studies, results are difficult to interpret. While some scales make theoretical sense to be correlated (e.g., functional scales: QLQ-C30 emotional functioning and QLQ-CR29 anxiety), many scales are likely unrelated (e.g., symptom scales: QLQ-C30 insomnia and QLQ-CR29 hair loss). Due to the diversity of subscale constructs, the results were rated as “indeterminate” in these studies.

**Table 5:** Convergent validity of the QLQ-CR29

Reference	Comparison instrument	Correlations	Quality	Rating
Arraras et al. [28]	QLQ-C30	C30 social functioning and CR29 body image, 0.57 C30 emotional functioning and CR29 body image, 0.51 C30 pain and CR39 abdominal pain, 0.51 C30 constipation and CR29 buttock pain, 0.52 C30 diarrhea and CR29 fecal incontinence, 0.51	Poor	Indeterminate
Hou et al. [29]	LARS score	Total LARS score and QLQ-CR29 flatulence, 0.49 Total LARS score and QLQ-CR29 fecal incontinence, 0.55 Total LARS score and QLQ-CR29 sore skin, 0.39 Total LARS score and QLQ-CR29 stool frequency, 0.57 Total LARS score and QLQ-CR29 embarrassment, 0.48	Good	Sufficient
Ihn et al. [30]	QLQ-C30	Correlations between subscales of the QLQ-CR29 and QLQ-C30 were low (*r* < 0.40) in most cases, whereas several areas with more related content showed higher correlations (*r* ≥ 0.40). Most functional subscales of the QLQ-CR29 were positively correlated with functional scales of the QLQ-C30, and negatively correlated with symptom scales of the QLQ-C30. In addition, most symptom scales were positively correlated with symptom scales of the QLQ-C30, and negatively correlated with functional scales.	Poor	Indeterminate
Lin et al. [31]	QLQ-C30	QLQ-C29 abdominal pain showed high correlations with QLQ-C30 pain (*r* = 0.65), and QLQ-C30 fatigue (*r* = 0.41). QLQ-CR29 anxiety showed high negative correlations with QLQ-C30 role functioning (r = −0.43), QLQ-C30 emotional functioning (*r* = − 0.55) and QLQ-C30 social functioning (*r* = − 0.41), and a positive correlation with QLQ-C30 financial problems (r = 0.45). QLQ-CR29 showed high correlations with QLQ-C30 blood and mucus in stool and QLQ-C30 quality of life (*r* = − 0.41), pain (*r* = 0.45), and diarrhea (*r* = 0.53). QLQ-CR29 body image, buttock pain, hair loss, bloating, fecal incontinence, sore skin and dyspareunia had correlation coefficients with QLQ-C30 nausea/vomiting that were higher than 0.4, and the correlations between the stool frequency and diarrhea as well as taste and appetite loss were also greater than 0.4.	Poor	Indeterminate
Magaji et al. [32]	QLQ-C30	Numerous significant correlations were observed between subscales of the QLQ-C30 and QLQ-CR29. These correlations were mostly weak (*r* ≤ 0.50). The strongest positive correlation was between QLQ-C30 financial difficulty and QLQ-CR29 stoma care problems (*r* = 0.71), and the strongest negative correlation was between QLQ-C30 emotional functioning and QLQ-CR29 stoma care problems (*r* = − 0.71).	Poor	Indeterminate
Montazeri et al. [33]	QLQ-C30	In general, functional scales of the QLQ-CR29 were positively correlated with the QLQ-C30 functional scales, and negatively correlated with QLQ-C30 symptom scales, and the QLQ-CR29 symptom scales were positively correlated with the QLQ-C30 symptom scales, and negatively correlated with the QLQ-C30 functional scales. These correlations were mostly weak (*r* < 0.40).	Poor	Indeterminate
Sanna et al. [35]	QLQ-C30	Most correlations between QLQ-C30 and QLQ-CR29 subscales were low (*r* < 0.40). The highest correlation was found between QLQ-C30 emotional functioning and QLQ-CR29 body image (*r* = 0.66). In general, the functional scales of the QLQ-CR29 were positively correlated with the QLQ-C30 functional scales, and negatively correlated with QLQ-C30 symptom scales, and the QLQ-CR29 symptom scales were positively correlated with the QLQ-C30 symptom scales, and negatively correlated with the QLQ-C30 functional scales.	Poor	Indeterminate
Stiggelbout et al. [37]	QLQ-C30	Correlations between the subscales of the QLQ-CR29 and QLQ-C30 were below 0.40, except for the subscales QLQ-CR29 body image and QLQ-C30 social functioning (*r* = 0.48), which correlated moderately.	Poor	Indeterminate

#### Divergent validity

Two studies investigated *divergent validity*. Methodological quality was rated as “poor” [11, 28], because a priori hypotheses about expected correlations were not reported, and information on the measurement properties of the comparator instrument was not provided. In both studies, the comparator instrument was the QLQ-C30 [6]. One study reported correlations between the two instruments of < 0.02 for most subscales [28], and the other reported correlations of < 0.40 in all subscales [11]. As was the case for convergent validity, due to the diversity of subscale constructs it is difficult to determine which subscales should be unrelated and which should be related. As such, we rated these results as “indeterminate.”

Based on these findings, *construct validity* (*hypothesis testing*) was rated overall as “inconsistent.” Most studies did not report a priori hypotheses, and therefore could not be interpreted. Three remaining studies provided an “insufficient” rating for known-group comparison [28, 36], and a “sufficient” rating for convergent validity [29], leading to the overall “inconsistent” rating. Quality of evidence was graded as “moderate,” because mostly studies of fair and poor quality were found.

### Responsiveness

Two studies investigated *responsiveness*. Methodological quality of these studies was rated as “poor” [11, 33], because a priori hypotheses about changes in scores were not reported. Sensitivity to measure change in HRQOL was tested in patients before and within 2 years after stoma closure, and in patients receiving palliative chemotherapy and 3 months later [11], and before and after neoadjuvant or palliative chemotherapy [33]. A statistically significant reduction was found of the symptoms scores on weight [11], and BMS, SF, urinary frequency, urinary incontinence, dysuria, buttock pain, bloating, and taste [33] after chemotherapy. Other scores were unchanged, as was the case after stoma closure. Based on these findings and the fact that no correlations with changes in instruments measuring related constructs were reported, the evidence on *responsiveness* was rated “indeterminate.”

Summarized ratings of the results and the overall quality of evidence of all measurement properties are shown in Table 6.

**Table 6:** Summary of results and quality of the evidence of the measurement properties of the QLQ-C29

Measurement property	Results	Quality of evidence
Content validity	NA	NA
Structural validity	Indeterminate	–
Internal consistency	Sufficient	Low
Reliability	Insufficient	Moderate
Measurement error	Indeterminate	–
Construct validity (hypothesis testing)	Inconsistent	Moderate
Known-group comparison	Sufficient	
Convergent validity	Insufficient	
Divergent validity	Indeterminate	
Cross-cultural validity	NA	NA
Responsiveness	Indeterminate	–

## Discussion

The QLQ-CR29 is a well-known and commonly used PROM, which was published in 2007, following revision of the QLQ-CR38. Both instruments cover a wide range of symptoms among CRC patients. This review shows that current evidence on the measurement properties of the QLQ-CR29 is limited. For each of the 11 studies included in the review, methodological quality per measurement property was rated most often as “fair” or “poor.” Evidence of *internal consistency* was rated as “sufficient,” *reliability* as “insufficient,” *construct validity* (*hypotheses testing*) as “inconsistent,” and *structural validity*, *measurement error*, and *responsiveness* as “indeterminate.”

Most studies performed indirect measurements of *structural validity*. With PCA, one of the original subscales could not be confirmed but was changed into a new subscale [37]. We recommend future studies to perform CFA, to confirm either the original or newly found factor structure. Subsequently, *internal consistency* should be assessed on those subscales that are confirmed to be unidimensional.

*Reliability* appears to be a concern for the QLQ-CR29. Further investigation is necessary, using ICC to control for systematic error variance. These data can also be used to assess *measurement error*, by calculating SDC. The SDC should be compared with the MIC, in order to determine whether the smallest change in scores that can be detected is smaller than the change that is minimally important for patients, and is not due, with 95% certainty, to measurement error.

*Criterion validity* cannot be assessed for the QLQ-CR29, since there is no “gold standard” for measuring HRQOL. Therefore, it is important to assess *construct validity* by formulating hypotheses, a priori, for (1) known-group differences and (2) assessing convergent and divergent validity with other PROMs, including direction and magnitude. Hypotheses that can be confirmed contribute to *construct validity*. The aim of the studies included in this review was primarily to determine whether there was overlap between the QLQ-C30 and QLQ-CR29, and not to specifically test for convergent/divergent validity. Therefore, *construct validity* of the QLQ-CR29 needs to be investigated further with a priori formulated hypotheses. The same applies to *responsiveness*, which needs to be investigated in groups that are known to change, with a priori formulated hypotheses.

While none of the studies reported on tests of *cross-cultural validity* (i.e., measurement invariance), the original validation study was performed in an international sample [11]. Additional, formal tests of measurement invariance would be useful.

The strength of the current review is that we closely followed the COSMIN guidelines during all steps of this review. A limitation is that we used a precise instead of a sensitive search filter for measurement properties in the literature search, which has a lower sensitivity (93 vs. 97%) [22]. Therefore, we cannot rule out that some additional validation studies of the QLQ-CR29 might have been missed.

This review indicates that additional, better quality research is needed on the measurement properties of the QLQ-CR29. Future validation studies should focus on assessing *structural validity* and subsequently *internal consistency* on subscales that are unidimensional, *reliability* and thereby *measurement error*, *construct validity* (*hypothesis testing*), and *responsiveness* with a priori hypotheses, and *cross-cultural validity.* It is thereby recommended to use the COSMIN methodology.

### Electronic supplementary material

ESM 1 (DOCX 169 kb)

ESM 2 (DOCX 39 kb)

## Appendix. Search terms

### Embase.com

(‘Perceived Stress Scale’/de OR ‘Insomnia Severity Index’/de OR ‘International Index of Erectile Function’/de OR ((cancer NEAR/3 worr* NEAR/3 scale*) OR (patient NEAR/3 specifieke NEAR/3 klacht*) OR (insomni* NEAR/3 sever* NEAR/3 index*) OR (6-item NEAR/6 female NEAR/3 sexual* NEAR/3 function*) OR (5-item NEAR/6 erectile NEAR/3 function*) OR (sexual* NEAR/3 health NEAR/3 inventor* NEAR/3 men) OR (body NEAR/3 image NEAR/3 scal*) OR ((EORTC OR ‘European Organization for Research and Treatment of Cancer’) NEAR/6 (QLQ OR ‘Quality of Life’) NEAR/6 (PATSAT32 OR BR23 OR BR-23 OR CR-29 OR CR29 OR H&N25 OR HN25 OR HN-25)) OR (Caron NEAR/3 screening NEAR/3 questionnaire*) OR (Jong NEAR/3 Gierveld NEAR/3 loneliness) OR (7-item NEAR/3 dyadis NEAR/3 adjustment*) OR (vragenlijst NEAR/3 gezinskenmerken) OR (job NEAR/3 content* NEAR/3 questionnaire*) OR (vragenlijst NEAR/3 beleving NEAR/3 beoordeling NEAR/3 arbeid) OR (Alcohol NEAR/3 five-shot) OR (perceived NEAR/3 stress NEAR/3 scale*) OR (functional NEAR/3 assessment NEAR/3 cancer NEAR/3 therap* NEAR/3 endocrine) OR (breast NEAR/3 impact NEAR/3 treatment NEAR/3 scale*) OR (breast NEAR/3 reconstruction NEAR/3 satisfaction NEAR/3 questionnair*) OR (breast NEAR/3 cancer NEAR/3 patients NEAR/3 needs NEAR/3 questionnaire*) OR (stoma NEAR/3 quality NEAR/3 life NEAR/3 questionnaire*) OR (shoulder* NEAR/3 disabilit* NEAR/3 questionnaire*) OR ((‘CWS’ OR ‘SPK’ OR ‘FSFI-6’ OR ‘IIEF-5’ OR ‘CARON’ OR ‘JGLS’ OR ‘DAS-7’ OR ‘VGK-SF’ OR ‘JCQ’ OR ‘VBBA’ OR ‘A5S’ OR ‘FACT-ES’ OR ‘BITS’ OR ‘BRECON-31’ OR ‘BR-CNPQ’ OR ‘SDQ’ OR ‘stoma-QoL’) NEAR/3 (assess* OR score* OR scale* OR questionnaire* OR inventor* OR measure*))):ab,ti) AND (neoplasm/exp OR (neoplas* OR cancer* OR oncolog* OR tumor* OR tumour OR carcino*):ab,ti) AND (‘validation study’/de OR ‘reproducibility’/de OR ‘psychometry’/de OR ‘observer variation’/de OR ‘discriminant analysis’/de OR ‘correlation coefficient’/de OR reliability/de OR ‘sensitivity and specificity’/de OR validity/exp OR ‘sensitivity analysis’/de OR ‘internal consistency’/de OR ‘confidence interval’/de OR (psychometr* OR reproducib* OR clinimetr* OR clinometr* OR observer-varia* OR reliab* OR valid* OR coefficient OR interna*-consisten* OR (cronbach* NEAR/3 (alpha OR alphas)) OR (item* NEXT/1 (correlation* OR selection* OR reduction*)) OR agreement OR precision OR imprecision OR precise-value* OR test*-retest* OR (test NEAR/3 retest) OR (reliab* NEAR/3 (test OR retest)) OR stability OR interrater OR inter-rater OR intrarater OR intra-rater OR intertester OR inter-tester OR intratester OR intra-tester OR interobserver OR inter-observer OR intraobserver OR intra-observer OR intertechnician OR inter-technician OR intratechnician OR intra-technician OR interexaminer OR inter-examiner OR intraexaminer OR intra-examiner OR interassay OR inter-assay OR intraassay OR intra-assay OR interindividual OR inter-individual OR intraindividual OR intra-individual OR interparticipant OR inter-participant OR intraparticipant OR intra-participant OR kappa OR kappa-s OR kappas OR (coefficient* NEAR/3 variation*) OR repeatab* OR ((replicab* OR repeat*) NEAR/3 (measure OR measures OR findings OR result OR results OR test OR tests)) OR generaliza* OR generalisa* OR concordance OR (intraclass NEAR/3 correlation*) OR discriminative OR ‘known group’ OR (factor* NEAR/3 (analys* OR structure*)) OR dimensionality OR subscale* OR (multitrait NEAR/3 scaling) OR item-discriminant* OR (interscale NEAR/3 correlat*) OR ((error OR errors) NEAR/3 (measure* OR correlat* OR evaluat* OR accuracy OR accurate OR precision OR mean)) OR ((individual OR interval OR rate OR analy*) NEAR/3 variabilit*) OR (uncertaint* NEAR/3 (measure*)) OR (error NEAR/3 measure*) OR sensitiv* OR responsive* OR (limit NEAR/3 detection) OR (minimal* NEAR/3 detectab*) OR interpretab* OR (small* NEAR/3 (real OR detectable) NEAR/3 (change OR difference)) OR (meaningful* NEAR/3 change*) OR (minimal* NEAR/3 (important OR detectab* OR real) NEAR/3 (change* OR difference)) OR ((ceiling OR floor) NEXT/1 effect*) OR ‘Item response model’ OR IRT OR Rasch OR ‘Differential item functioning’ OR DIF OR ‘computer adaptive testing’ OR ‘item bank’ OR ‘cross-cultural equivalence’ OR (confidence* NEAR/3 interval*)):ab,ti)

### Medline Ovid

(((cancer ADJ3 worr* ADJ3 scale*) OR (patient ADJ3 specifieke ADJ3 klacht*) OR (insomni* ADJ3 sever* ADJ3 index*) OR (6-item ADJ6 female ADJ3 sexual* ADJ3 function*) OR (5-item ADJ6 erectile ADJ3 function*) OR (sexual* ADJ3 health ADJ3 inventor* ADJ3 men) OR (body ADJ3 image ADJ3 scal*) OR ((EORTC OR “European Organization for Research and Treatment of Cancer”) ADJ6 (QLQ OR “Quality of Life”) ADJ6 (PATSAT32 OR BR23 OR BR-23 OR CR-29 OR CR29 OR H&N25 OR HN25 OR HN-25)) OR (Caron ADJ3 screening ADJ3 questionnaire*) OR (Jong ADJ3 Gierveld ADJ3 loneliness) OR (7-item ADJ3 dyadis ADJ3 adjustment*) OR (vragenlijst ADJ3 gezinskenmerken) OR (job ADJ3 content* ADJ3 questionnaire*) OR (vragenlijst ADJ3 beleving ADJ3 beoordeling ADJ3 arbeid) OR (Alcohol ADJ3 five-shot) OR (perceived ADJ3 stress ADJ3 scale*) OR (functional ADJ3 assessment ADJ3 cancer ADJ3 therap* ADJ3 endocrine) OR (breast ADJ3 impact ADJ3 treatment ADJ3 scale*) OR (breast ADJ3 reconstruction ADJ3 satisfaction ADJ3 questionnair*) OR (breast ADJ3 cancer ADJ3 patients ADJ3 needs ADJ3 questionnaire*) OR (stoma ADJ3 quality ADJ3 life ADJ3 questionnaire*) OR (shoulder* ADJ3 disabilit* ADJ3 questionnaire*) OR ((“CWS” OR “SPK” OR “FSFI-6” OR “IIEF-5” OR “CARON” OR “JGLS” OR “DAS-7” OR “VGK-SF” OR “JCQ” OR “VBBA” OR “A5S” OR “FACT-ES” OR “BITS” OR “BRECON-31” OR “BR-CNPQ” OR “SDQ” OR “stoma-QoL”) ADJ3 (assess* OR score* OR scale* OR questionnaire* OR inventor* OR measure*))).ab,ti.) AND (neoplasm/ OR (neoplas* OR cancer* OR oncolog* OR tumor* OR tumour OR carcino*).ab,ti.) AND (exp “Validation Studies”/ OR exp “reproducibility of results”/ OR exp “psychometrics”/ OR exp “observer variation”/ OR exp “discriminant analysis”/ OR exp “Sensitivity and Specificity”/ OR “Confidence Intervals”/ OR (psychometr* OR reproducib* OR clinimetr* OR clinometr* OR observer-varia* OR reliab* OR valid* OR coefficient OR interna*-consisten* OR (cronbach* ADJ3 (alpha OR alphas)) OR (item* ADJ (correlation* OR selection* OR reduction*)) OR agreement OR precision OR imprecision OR precise-value* OR test*-retest* OR (test ADJ3 retest) OR (reliab* ADJ3 (test OR retest)) OR stability OR interrater OR inter-rater OR intrarater OR intra-rater OR intertester OR inter-tester OR intratester OR intra-tester OR interobserver OR inter-observer OR intraobserver OR intra-observer OR intertechnician OR inter-technician OR intratechnician OR intra-technician OR interexaminer OR inter-examiner OR intraexaminer OR intra-examiner OR interassay OR inter-assay OR intraassay OR intra-assay OR interindividual OR inter-individual OR intraindividual OR intra-individual OR interparticipant OR inter-participant OR intraparticipant OR intra-participant OR kappa OR kappa-s OR kappas OR (coefficient* ADJ3 variation*) OR repeatab* OR ((replicab* OR repeat*) ADJ3 (measure OR measures OR findings OR result OR results OR test OR tests)) OR generaliza* OR generalisa* OR concordance OR (intraclass ADJ3 correlation*) OR discriminative OR “known group” OR (factor* ADJ3 (analys* OR structure*)) OR dimensionality OR subscale* OR (multitrait ADJ3 scaling) OR item-discriminant* OR (interscale ADJ3 correlat*) OR ((error OR errors) ADJ3 (measure* OR correlat* OR evaluat* OR accuracy OR accurate OR precision OR mean)) OR ((individual OR interval OR rate OR analy*) ADJ3 variabilit*) OR (uncertaint* ADJ3 (measure*)) OR (error ADJ3 measure*) OR sensitiv* OR responsive* OR (limit ADJ3 detection) OR (minimal* ADJ3 detectab*) OR interpretab* OR (small* ADJ3 (real OR detectable) ADJ3 (change OR difference)) OR (meaningful* ADJ3 change*) OR (minimal* ADJ3 (important OR detectab* OR real) ADJ3 (change* OR difference)) OR ((ceiling OR floor) ADJ effect*) OR “Item response model” OR IRT OR Rasch OR “Differential item functioning” OR DIF OR “computer adaptive testing” OR “item bank” OR “cross-cultural equivalence” OR (confidence* ADJ3 interval*)).ab,ti.)

### PsycINFO Ovid

(((cancer ADJ3 worr* ADJ3 scale*) OR (patient ADJ3 specifieke ADJ3 klacht*) OR (insomni* ADJ3 sever* ADJ3 index*) OR (6-item ADJ6 female ADJ3 sexual* ADJ3 function*) OR (5-item ADJ6 erectile ADJ3 function*) OR (sexual* ADJ3 health ADJ3 inventor* ADJ3 men) OR (body ADJ3 image ADJ3 scal*) OR ((EORTC OR “European Organization for Research and Treatment of Cancer”) ADJ6 (QLQ OR “Quality of Life”) ADJ6 (PATSAT32 OR BR23 OR BR-23 OR CR-29 OR CR29 OR H&N25 OR HN25 OR HN-25)) OR (Caron ADJ3 screening ADJ3 questionnaire*) OR (Jong ADJ3 Gierveld ADJ3 loneliness) OR (7-item ADJ3 dyadis ADJ3 adjustment*) OR (vragenlijst ADJ3 gezinskenmerken) OR (job ADJ3 content* ADJ3 questionnaire*) OR (vragenlijst ADJ3 beleving ADJ3 beoordeling ADJ3 arbeid) OR (Alcohol ADJ3 five-shot) OR (perceived ADJ3 stress ADJ3 scale*) OR (functional ADJ3 assessment ADJ3 cancer ADJ3 therap* ADJ3 endocrine) OR (breast ADJ3 impact ADJ3 treatment ADJ3 scale*) OR (breast ADJ3 reconstruction ADJ3 satisfaction ADJ3 questionnair*) OR (breast ADJ3 cancer ADJ3 patients ADJ3 needs ADJ3 questionnaire*) OR (stoma ADJ3 quality ADJ3 life ADJ3 questionnaire*) OR (shoulder* ADJ3 disabilit* ADJ3 questionnaire*) OR ((“CWS” OR “SPK” OR “FSFI-6” OR “IIEF-5” OR “CARON” OR “JGLS” OR “DAS-7” OR “VGK-SF” OR “JCQ” OR “VBBA” OR “A5S” OR “FACT-ES” OR “BITS” OR “BRECON-31” OR “BR-CNPQ” OR “SDQ” OR “stoma-QoL”) ADJ3 (assess* OR score* OR scale* OR questionnaire* OR inventor* OR measure*))).ab,ti.) AND (neoplasm/ OR (neoplas* OR cancer* OR oncolog* OR tumor* OR tumour OR carcino*).ab,ti.) AND (exp “Test Validity”/ OR exp “Test Reliability”/ OR exp “psychometrics”/ OR exp “Interrater Reliability”/ OR exp OR (psychometr* OR reproducib* OR clinimetr* OR clinometr* OR observer-varia* OR reliab* OR valid* OR coefficient OR interna*-consisten* OR (cronbach* ADJ3 (alpha OR alphas)) OR (item* ADJ (correlation* OR selection* OR reduction*)) OR agreement OR precision OR imprecision OR precise-value* OR test*-retest* OR (test ADJ3 retest) OR (reliab* ADJ3 (test OR retest)) OR stability OR interrater OR inter-rater OR intrarater OR intra-rater OR intertester OR inter-tester OR intratester OR intra-tester OR interobserver OR inter-observer OR intraobserver OR intra-observer OR intertechnician OR inter-technician OR intratechnician OR intra-technician OR interexaminer OR inter-examiner OR intraexaminer OR intra-examiner OR interassay OR inter-assay OR intraassay OR intra-assay OR interindividual OR inter-individual OR intraindividual OR intra-individual OR interparticipant OR inter-participant OR intraparticipant OR intra-participant OR kappa OR kappa-s OR kappas OR (coefficient* ADJ3 variation*) OR repeatab* OR ((replicab* OR repeat*) ADJ3 (measure OR measures OR findings OR result OR results OR test OR tests)) OR generaliza* OR generalisa* OR concordance OR (intraclass ADJ3 correlation*) OR discriminative OR “known group” OR (factor* ADJ3 (analys* OR structure*)) OR dimensionality OR subscale* OR (multitrait ADJ3 scaling) OR item-discriminant* OR (interscale ADJ3 correlat*) OR ((error OR errors) ADJ3 (measure* OR correlat* OR evaluat* OR accuracy OR accurate OR precision OR mean)) OR ((individual OR interval OR rate OR analy*) ADJ3 variabilit*) OR (uncertaint* ADJ3 (measure*)) OR (error ADJ3 measure*) OR sensitiv* OR responsive* OR (limit ADJ3 detection) OR (minimal* ADJ3 detectab*) OR interpretab* OR (small* ADJ3 (real OR detectable) ADJ3 (change OR difference)) OR (meaningful* ADJ3 change*) OR (minimal* ADJ3 (important OR detectab* OR real) ADJ3 (change* OR difference)) OR ((ceiling OR floor) ADJ effect*) OR “Item response model” OR IRT OR Rasch OR “Differential item functioning” OR DIF OR “computer adaptive testing” OR “item bank” OR “cross-cultural equivalence” OR (confidence* ADJ3 interval*)).ab,ti.)

### Web of science

TS = ((((cancer NEAR/3 worr* NEAR/3 scale*) OR (patient NEAR/3 specifieke NEAR/3 klacht*) OR (insomni* NEAR/3 sever* NEAR/3 index*) OR (6-item NEAR/6 female NEAR/3 sexual* NEAR/3 function*) OR (5-item NEAR/6 erectile NEAR/3 function*) OR (sexual* NEAR/3 health NEAR/3 inventor* NEAR/3 men) OR (body NEAR/3 image NEAR/3 scal*) OR ((EORTC OR “European Organization for Research and Treatment of Cancer”) NEAR/6 (QLQ OR “Quality of Life”) NEAR/6 (PATSAT32 OR BR23 OR BR-23 OR CR-29 OR CR29 OR H&N25 OR HN25 OR HN-25)) OR (Caron NEAR/3 screening NEAR/3 questionnaire*) OR (Jong NEAR/3 Gierveld NEAR/3 loneliness) OR (7-item NEAR/3 dyadis NEAR/3 adjustment*) OR (vragenlijst NEAR/3 gezinskenmerken) OR (job NEAR/3 content* NEAR/3 questionnaire*) OR (vragenlijst NEAR/3 beleving NEAR/3 beoordeling NEAR/3 arbeid) OR (Alcohol NEAR/3 five-shot) OR (perceived NEAR/3 stress NEAR/3 scale*) OR (functional NEAR/3 assessment NEAR/3 cancer NEAR/3 therap* NEAR/3 endocrine) OR (breast NEAR/3 impact NEAR/3 treatment NEAR/3 scale*) OR (breast NEAR/3 reconstruction NEAR/3 satisfaction NEAR/3 questionnair*) OR (breast NEAR/3 cancer NEAR/3 patients NEAR/3 needs NEAR/3 questionnaire*) OR (stoma NEAR/3 quality NEAR/3 life NEAR/3 questionnaire*) OR (shoulder* NEAR/3 disabilit* NEAR/3 questionnaire*) OR ((“CWS” OR “SPK” OR “FSFI-6” OR “IIEF-5” OR “CARON” OR “JGLS” OR “DAS-7” OR “VGK-SF” OR “JCQ” OR “VBBA” OR “A5S” OR “FACT-ES” OR “BITS” OR “BRECON-31” OR “BR-CNPQ” OR “SDQ” OR “stoma-QoL”) NEAR/3 (assess* OR score* OR scale* OR questionnaire* OR inventor* OR measure*)))) AND (neoplasm/exp OR (neoplas* OR cancer* OR oncolog* OR tumor* OR tumour OR carcino*)) AND ((psychometr* OR reproducib* OR clinimetr* OR clinometr* OR observer-varia* OR reliab* OR valid* OR coefficient OR interna*-consisten* OR (cronbach* NEAR/3 (alpha OR alphas)) OR (item* NEAR/1 (correlation* OR selection* OR reduction*)) OR agreement OR precision OR imprecision OR precise-value* OR test*-retest* OR (test NEAR/3 retest) OR (reliab* NEAR/3 (test OR retest)) OR stability OR interrater OR inter-rater OR intrarater OR intra-rater OR intertester OR inter-tester OR intratester OR intra-tester OR interobserver OR inter-observer OR intraobserver OR intra-observer OR intertechnician OR inter-technician OR intratechnician OR intra-technician OR interexaminer OR inter-examiner OR intraexaminer OR intra-examiner OR interassay OR inter-assay OR intraassay OR intra-assay OR interindividual OR inter-individual OR intraindividual OR intra-individual OR interparticipant OR inter-participant OR intraparticipant OR intra-participant OR kappa OR kappa-s OR kappas OR (coefficient* NEAR/3 variation*) OR repeatab* OR ((replicab* OR repeat*) NEAR/3 (measure OR measures OR findings OR result OR results OR test OR tests)) OR generaliza* OR generalisa* OR concordance OR (intraclass NEAR/3 correlation*) OR discriminative OR “known group” OR (factor* NEAR/3 (analys* OR structure*)) OR dimensionality OR subscale* OR (multitrait NEAR/3 scaling) OR item-discriminant* OR (interscale NEAR/3 correlat*) OR ((error OR errors) NEAR/3 (measure* OR correlat* OR evaluat* OR accuracy OR accurate OR precision OR mean)) OR ((individual OR interval OR rate OR analy*) NEAR/3 variabilit*) OR (uncertaint* NEAR/3 (measure*)) OR (error NEAR/3 measure*) OR sensitiv* OR responsive* OR (limit NEAR/3 detection) OR (minimal* NEAR/3 detectab*) OR interpretab* OR (small* NEAR/3 (real OR detectable) NEAR/3 (change OR difference)) OR (meaningful* NEAR/3 change*) OR (minimal* NEAR/3 (important OR detectab* OR real) NEAR/3 (change* OR difference)) OR ((ceiling OR floor) NEAR/1 effect*) OR “Item response model” OR IRT OR Rasch OR “Differential item functioning” OR DIF OR “computer adaptive testing” OR “item bank” OR “cross-cultural equivalence” OR (confidence* NEAR/3 interval*))))

## Abbreviations

BI: Body image
BMS: Blood and mucus in stool
COSMIN: COnsensus-based Standards for the selection of health Measurement INstruments
CFA: Confirmatory factor analysis
CRC: Colorectal cancer
DSP: Defecation/stoma problems
EFA: Exploratory factor analysis
EORTC: European Organization for Research and Treatment of Cancer
(EORTC) QLQ-C30: EORTC Quality of Life Questionnaire, 30-item core questionnaire
(EORTC) QLQ-CR29: EORTC Quality of Life Questionnaire, 29-item colorectal specific questionnaire
EORTC QLQ-CR38: EORTC Quality of Life Questionnaire, 38-item colorectal specific questionnaire
FACT-C: Functional Assessment of Cancer Therapy-Colorectal
GRADE: Grades of Recommendation, Assessment, Development and Evaluation
HRQOL: Health-related quality Of life
ICC: Intraclass correlation coefficient
KPS: Karnofsky performance score
MIC: Minimal important change
MIS: Multitrait item scaling
LARS: Low anterior resection syndrome
PCA: Principal component analysis
PRISMA: Preferred Reporting Items for Systematic Reviews and Meta-Analyses
PROM: Patient-reported outcome measure
SDC: Smallest detectable change
SEM: Standard error of measurement
SF: Stool frequency
UF: Urinary frequency
