# Supplementary material for: Measuring health-related quality of life in colorectal cancer patients: systematic review of measurement properties of the EORTC QLQ-CR29
Source: Support Care Cancer. 2019 Apr 13;27(7):2395–412. doi: 10.1007/s00520-019-04764-7 (PMC6541702; doi:10.1007/s00520-019-04764-7)
Supplement: Supplementary file 1 — (DOCX 169 kb) [file 520_2019_4764_MOESM1_ESM.docx]

**
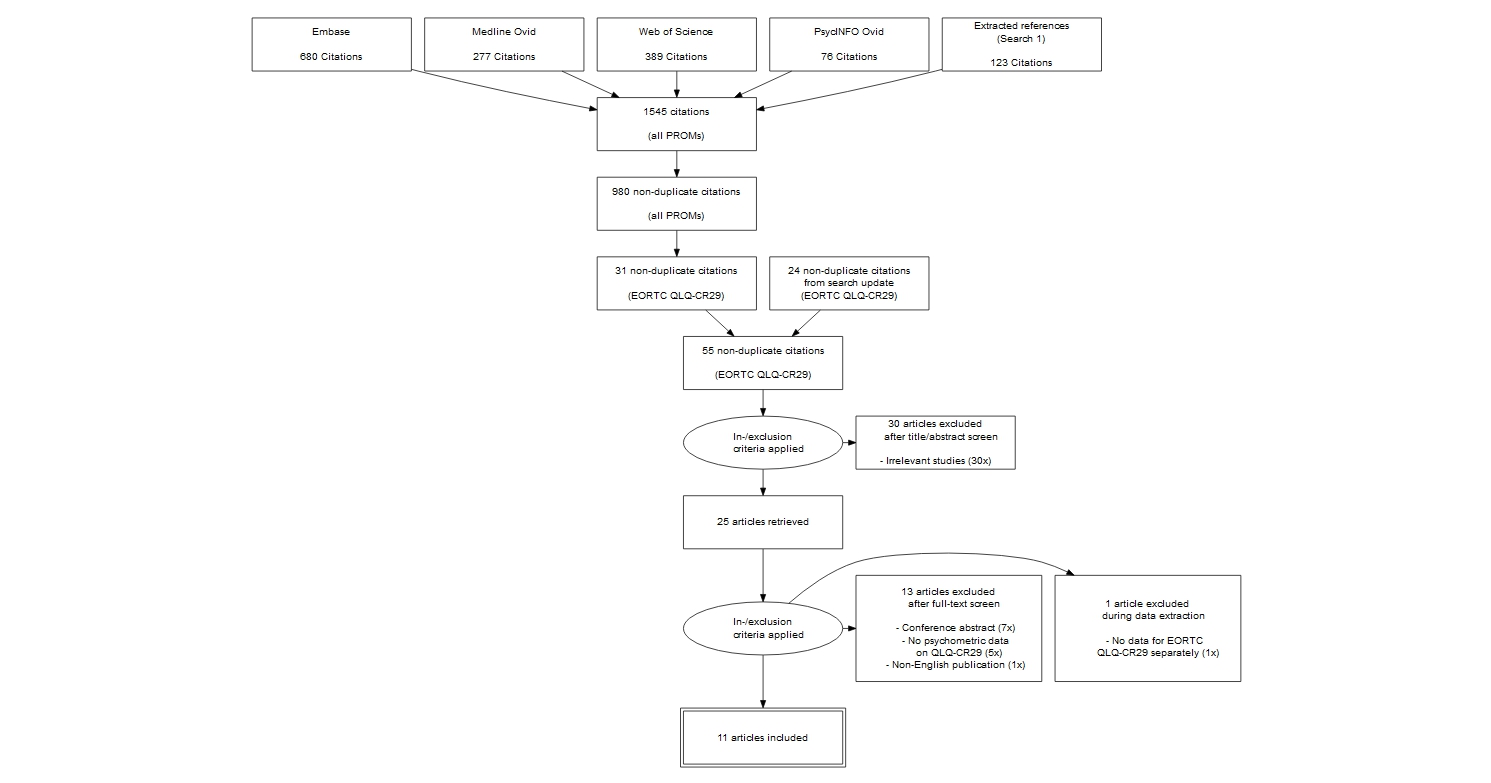
**

**Supplementary Figure 1** – Preferred Reporting Items for Systematic Reviews and Meta-Analyses (PRISMA) flow diagram of the literature search and selection
