# Supplementary material for: Measuring health-related quality of life in colorectal cancer patients: systematic review of measurement properties of the EORTC QLQ-CR29
Source: Support Care Cancer. 2019 Apr 13;27(7):2395–412. doi: 10.1007/s00520-019-04764-7 (PMC6541702; doi:10.1007/s00520-019-04764-7)
Supplement: Supplementary file 2 — (DOCX 39 kb) [file 520_2019_4764_MOESM2_ESM.docx]

**Supplementary Table 1** – Internal consistency (Cronbach’s α) of the QLQ-CR29 subscales

| **Reference** | **Sample** | **UF** | **BMS** | **SF** | **BI** | **DSP** | **Quality** | **Rating** |
| --- | --- | --- | --- | --- | --- | --- | --- | --- |
| Arraras et al. [28] |  |  |  |  |  |  | Poor | Indeterminate |
|  | Total sample | 0.70 | 0.27 | 0.64 | 0.68 | - |  |  |
| Ihn et al. [30] |  |  |  |  |  |  | Poor | Indeterminate |
|  | Total sample | 0.70 | 0.72 | 0.80 | 0.87 | - |  |  |
| Lin et al. [31] |  |  |  |  |  |  | Poor | Indeterminate |
|  | Total sample | 0.41 | 0.64 | 0.68 | 0.72 | - |  |  |
|  | Patients with stoma | 0.36 | 0.64 | 0.72 | 0.72 | - |  |  |
|  | Patients without stoma | 0.61 | 0.63 | 0.42 | 0.68 | - |  |  |
| Magaji et al. [32] |  |  |  |  |  |  | Poor | Indeterminate |
|  | Total sample | 0.74 | 0.66 | 0.75 | 0.78 | - |  |  |
|  | Patients with stoma | 0.74 | 0.49 | 0.46 | 0.81 | - |  |  |
|  | Patients without stoma | 0.74 | 0.76 | 0.87 | 0.77 | - |  |  |
| Montazeri et al. [33] |  |  |  |  |  |  | Poor | Indeterminate |
|  | Total sample | 0.86 | 0.48 | 0.77 | 0.75 | - |  |  |
| Nowak et al. [34] |  |  |  |  |  |  | Poor | Indeterminate |
|  | Total sample | 0.66 | - | 0.74 | 0.83 | - |  |  |
|  | Patients with stoma | 0.69 | - | 0.75 | 0.63 | - |  |  |
|  | Patients without stoma | 0.66 | - | - | 0.55 | - |  |  |
| Sanna et al. [35] |  |  |  |  |  |  | Poor | Indeterminate |
|  | Total sample | 0.72 | 0.70 | - | 0.83 | 0.85 |  |  |
|  | Patients with stoma | - | - | 0.83 | - | 0.87 |  |  |
|  | Patients without stoma | - | - | 0.80 | - | 0.89 |  |  |
| Shen et al. [36] |  |  |  |  |  |  | Poor | Indeterminate |
|  | Total sample | 0.82 | 0.78 | 0.70 | 0.90 | - |  |  |
| Stiggelbout et al. [37] |  |  |  |  |  |  | Fair | Sufficient |
|  | Total sample | 0.71 | 0.56 | - | 0.80 | 0.84 |  |  |
|  | Patients with stoma | - | - | 0.72 | - | - |  |  |
|  | Patients without stoma | - | - | 0.68 | - | - |  |  |
| Whistance et al. [11] |  |  |  |  |  |  | Poor | Indeterminate |
|  | Total sample | 0.75 | 0.69 | 0.70 | 0.84 | - |  |  |
|  | Patients with stoma | 0.80 | 0.54 | 0.78 | 0.82 | - |  |  |
|  | Patients without stoma | 0.71 | 0.72 | 0.66 | 0.83 | - |  |  |

UF = Urinary frequency; BMS = Blood and mucus in stool; SF = Stool frequency; BI = Body image; DSP = Defaecation/stoma problems

**Supplementary Table 2** – Measurement error (Standard Error of Measurement [SEM] and Smallest Detectable Change [SDC]) of the QLQ-CR29

| **Reference** |  | **UF** | **BMS** | **SF** | **BI** | **DSP** | **UI** | **DY** | **AP** | **BP** | **BF** | **DM** | **HL** | **TA** | **ANX** |
| --- | --- | --- | --- | --- | --- | --- | --- | --- | --- | --- | --- | --- | --- | --- | --- |
| Ihn et al. [30] | |  |  |  |  |  |  |  |  |  |  |  |  |  |  |
|  | SEM | 10.38 | - | 6.37 | 5.12 | - | 0 | 3.39 | 6.58 | 4.04 | 9.52 | 9.26 | 3.82 | 8.35 | 10.60 |
|  | SDC | 28.77 | - | 17.65 | 14.20 | - | 0 | 9.41 | 18.25 | 11.20 | 26.39 | 25.66 | 10.60 | 23.14 | 29.37 |
| Sanna et al. [35] | |  |  |  |  |  |  |  |  |  |  |  |  |  |  |
|  | SEM | 13.00 | 4.54 | 8.13 | 7.44 | 6.96 | 16.36 | 10.85 | 8.79 | 7.23 | 9.49 | 9.98 | 4.64 | 8.39 | 14.84 |
|  | SDC | 36.03 | 12.58 | 22.53 | 20.62 | 19.31 | 45.35 | 30.07 | 24.37 | 20.04 | 26.30 | 27.67 | 12.86 | 23.25 | 41.14 |
| Shen et al. [36] | |  |  |  |  |  |  |  |  |  |  |  |  |  |  |
|  | SEM | 14.98 | 15.52 | 9.76 | 6.37 | - | 16.42 | 4.96 | 12.22 | 9.37 | 17.74 | 19.27 | 11.12 | 12.41 | 17.62 |
|  | SDC | 41.52 | 43.01 | 27.04 | 16.65 | - | 45.50 | 13.74 | 33.87 | 25.97 | 49.17 | 53.41 | 30.81 | 31.39 | 48.83 |
| Stiggelbout et al. [37] | |  |  |  |  |  |  |  |  |  |  |  |  |  |  |
|  | SEM | - | - | - | - | - | 16.28 | 10.96 | 10.17 | 12.65 | 15.23 | 6.80 | 8.87 | 12.45 | 17.91 |
|  | SDC | - | - | - | - | - | 45.12 | 30.38 | 28.20 | 35.05 | 42.21 | 18.85 | 24.58 | 34.51 | 49.63 |

**Supplementary Table 2** – continued

| **Reference** | | **WEI** | **FL** | **FI** | **SS** | **EMB** | **STO** | **IMP** | **DYS** | **SEXM** | **SEXW** | **Quality** | **Rating** |
| --- | --- | --- | --- | --- | --- | --- | --- | --- | --- | --- | --- | --- | --- |
| Ihn et al. [30] | |  |  |  |  |  |  |  |  |  |  | Fair | Indeterminate |
|  | SEM | 3.82 | 10.63 | 8.20 | 6.85 | 10.16 | 7.41 | 8.27 | - | 9.99 | - |  |  |
|  | SDC | 10.59 | 29.48 | 22.74 | 18.99 | 28.15 | 20.54 | 22.93 | - | 27.69 | - |  |  |
| Sanna et al. [35] | |  |  |  |  |  |  |  |  |  |  | Fair | Indeterminate |
|  | SEM | 11.29 | 9.16 | 8.72 | 7.69 | 10.37 | 7.41 | 14.80 | 11.85 | 8.23 | 8.89 |  |  |
|  | SDC | 31.28 | 25.40 | 24.17 | 21.32 | 28.76 | 20.54 | 41.03 | 32.83 | 22.81 | 24.65 |  |  |
| Shen et al. [36] | |  |  |  |  |  |  |  |  |  |  | Poor | Indeterminate |
|  | SEM | 17.52 | 11.33 | 14.56 | 10.10 | 17.11 | 4.15 | 3.72 | 11.95 | 14.41 ^b^ | 14.41 ^b^ |  |  |
|  | SDC | 48.57 | 31.41 | 40.36 | 27.99 | 47.43 | 11.49 | 10.32 | 33.13 | 39.96 ^b^ | 39.96 ^b^ |  |  |
| Stiggelbout et al. [37] | |  |  |  |  |  |  |  |  |  |  | Poor | Indeterminate |
|  | SEM | 14.32 | 16.44 | 11.6 | 11.37 | 17.75 | 7.41 | 19.56 ^a^ | 19.56 ^a^ | 9.84 ^b^ | 9.84 ^b^ |  |  |
|  | SDC | 39.71 | 45.57 | 32.15 | 31.52 | 49.20 | 20.54 | 54.21 ^a^ | 54.21 ^a^ | 27.27 ^b^ | 27.27 ^b^ |  |  |

Subscales: UF = Urinary frequency; BMS = Blood and mucus in stool; SF = Stool frequency; BI = Body image; DSP = Defaecation/Stoma Problems
Single items: UI = Urinary incontinence; DY = Dysuria; AP = Abdominal pain; BP = Buttock pain; BF = Bloating; DM = Dry mouth; HL = Hair loss; TA = Taste; ANX = Anxiety; WEI = Weight; FL = Flatulence; FI = Faecal incontinence; SS = Sore skin; EMB = Embarrassment; STO = Stoma care problems; IMP = Impotence; DYS = Dyspareunia; SEXM = Sexual interest (men); SEMW = Sexual interest (women)
^a^ values for IMP and DYS combined, ^b^ values for SEXM and SEXW combined
